# ASSOCIATION BETWEEN NEIGHBORHOOD DISADVANTAGE AND COGNITIVE RESERVE IN RACIALLY DIVERSE OLDER ADULTS

**DOI:** 10.1093/geroni/igad104.0172

**Published:** 2023-12-21

**Authors:** Ketlyne Sol, Philippa Clarke, Laura Zahodne

**Affiliations:** University of Michigan, Ann Arbor, Michigan, United States; University of Michigan, Ann Arbor, Michigan, United States; University of Michigan, Ann Arbor, Michigan, United States

## Abstract

Social determinants of health such as neighborhood disadvantage (NDis) are increasingly implicated in cognitive aging. More study is needed on the role of neighborhoods in cognitive reserve—better-than-expected cognitive function given brain health—and dementia disparities. This cross-sectional study included 98 non-Hispanic Black (NHB) and White (NHW) participants (mean age=62.7; 30% NHB) from the Michigan Cognitive Aging Project, a community-based study of adults transitioning to late life in the Detroit area. Participants’ current addresses were geocoded and linked to 2017 measures of NDis at the census tract level from the National Neighborhood Data Archive. NDis is a composite of four indicators of disadvantage for the 26 unique census tracts in the sample. Cognitive reserve was the residual of an episodic memory factor score after regressing out hippocampal volume, cortical thickness across nine Alzheimer’s disease signature regions, and education. Linear regression estimated the association between NDis and cognitive reserve, adjusting for age, sex/gender, and race. Analyses were repeated stratifying by race. In the entire sample, NDis was not associated with cognitive reserve (beta= -.08, p=.841), but stratified analyses indicated that more NDis was marginally associated with less cognitive reserve within NHB (beta= -.36, p=.057) but not NHW participants. NDis may contribute to cognitive reserve more strongly among NHB because of racially-patterned risk and resilience factors. Results highlight the need for more research into specific neighborhood factors that facilitate and deplete cognitive reserve in racially diverse older adults. Cognitive reserve may reflect contextual factors in addition to individual factors.

